# Plasminogen Activator Inhibitor-1 as a Therapeutic Target for Healthy Longevity, Immunosenescence, and Age-Related Disease: Translational Development of the Small-Molecule Inhibitor TM5614

**DOI:** 10.3390/cells15100941

**Published:** 2026-05-20

**Authors:** Mohamed Abdelhakim, Toshio Miyata

**Affiliations:** Department of Molecular Medicine and Therapy, Tohoku University Graduate School of Medicine, 2-1 Seiryo-machi, Aoba-ku, Sendai 980-8575, Japan; toshio.miyata.c8@tohoku.ac.jp

**Keywords:** PAI-1, SERPINE1, TM5614, fibrinolysis, immunoaging, inflammaging, cellular senescence, immune checkpoint, fibrosis, cancer microenvironment, longevity

## Abstract

Plasminogen activator inhibitor-1 (PAI-1), encoded by SERPINE1, is the principal physiological inhibitor of tissue-type and urokinase-type plasminogen activators and a central regulator of fibrinolysis. Beyond its canonical hemostatic role, PAI-1 has emerged as a pleiotropic mediator of tissue remodeling, fibrosis, metabolic dysfunction, cancer progression, cellular senescence, and age-associated immune dysregulation. A central argument of this review is that PAI-1 should be understood not only as a downstream biomarker of aging-associated pathology, but also as an active effector linking senescence-associated secretory phenotype (SASP) signaling, chronic low-grade inflammation, impaired immune surveillance, fibrotic extracellular matrix remodeling, and a prothrombotic state. In this framework, PAI-1 may function as an immune-aging checkpoint: a molecular node through which senescent, stromal, malignant, and inflammatory cells reinforce immune evasion and tissue dysfunction. Structure-guided drug discovery has enabled the development of small-molecule PAI-1 inhibitors, including TM5275, TM5441, TM5509, and TM5614. Among these, TM5614 is an orally available investigational compound that has progressed to clinical evaluation. Preclinical studies support anti-thrombotic, anti-fibrotic, anti-inflammatory, anti-senescent, and tumor-microenvironment-modulating effects of PAI-1 inhibition, while early clinical studies have evaluated TM5614 in chronic myeloid leukemia, immune-checkpoint-refractory malignant melanoma, non-small-cell lung cancer, and COVID-19-associated pneumonia. This review summarizes the biology of PAI-1, expands the discussion of immunoaging, reviews representative preclinical and clinical data, compares available PAI-1 inhibitors, and discusses the translational opportunities and safety considerations for TM5614 and related compounds.

## 1. Introduction

The plasminogen activation system maintains vascular and tissue homeostasis by regulating fibrinolysis, extracellular matrix turnover, wound repair, inflammatory-cell trafficking, and tissue remodeling. Plasminogen is converted into the active protease plasmin by tissue-type plasminogen activator (tPA) and urokinase-type plasminogen activator (uPA). Plasmin degrades fibrin and contributes to extracellular matrix proteolysis, thereby facilitating vascular repair and tissue remodeling [1,2].

PAI-1 forms inhibitory complexes with tPA and uPA, limiting plasmin generation and preserving the balance between coagulation and fibrinolysis. Although this function is essential for hemostatic control, persistently elevated PAI-1 creates a biological environment characterized by impaired fibrin clearance, extracellular matrix accumulation, vascular dysfunction, inflammatory persistence, and impaired regeneration [2,3,4].

The principal argument of this review is that PAI-1 is not merely a fibrinolytic inhibitor but a biological checkpoint connecting three major domains of age-related disease: (i) cellular senescence and SASP amplification, (ii) immune aging and immune evasion, and (iii) tissue remodeling with fibrosis and thrombosis. This expanded interpretation is important because diseases such as cancer, idiopathic and secondary fibrotic disorders, metabolic syndrome, cardiovascular disease, chronic infection-related inflammation, and degenerative aging phenotypes often coexist in older adults and share common inflammatory and stromal mechanisms.

Immunoaging refers to age-associated remodeling of the immune system, including reduced adaptive immune diversity, chronic innate immune activation, impaired tissue repair, diminished clearance of senescent or transformed cells, and sustained low-grade inflammation often described as inflammaging [5,6]. Senescent cells accumulate with age and secrete SASP factors such as inflammatory cytokines, chemokines, growth factors, matrix remodeling proteins, and protease regulators. PAI-1 is repeatedly identified as both a marker and mediator of senescence, making it a plausible mechanistic bridge between immunoaging, tissue fibrosis, and chronic disease [3,7,8,9,10,11,12].

**Figure 1:**
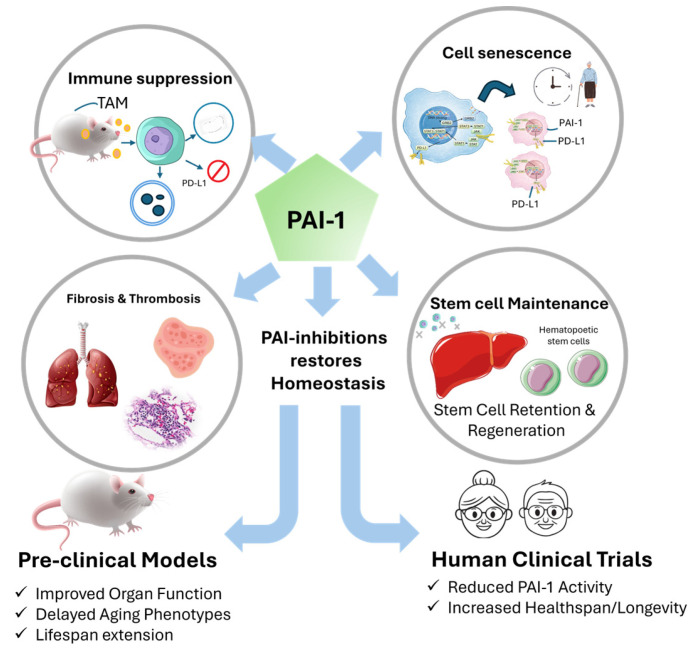
PAI-1 as a central hub linking cellular senescence, immune evasion, fibrosis, thrombosis, and systemic aging. High PAI-1 expression characterizes senescent and pathologically remodeled tissues, where it contributes to SASP amplification, PD-L1-associated immune evasion, stromal remodeling, pro-fibrotic extracellular matrix accumulation, and impaired fibrinolysis. Reduced PAI-1 activity in experimental and human genetic settings is associated with improved tissue function and healthspan-related phenotypes.

Recent work also indicates that senescent cells may acquire immune-evasive features analogous to malignant cells, including upregulation of immune checkpoint molecules such as PD-L1 [13]. PAI-1-rich tissue microenvironments can promote macrophage recruitment, fibroblast activation, extracellular matrix remodeling, stem-cell niche retention, and impaired immune-mediated clearance [13,14,15,16,17]. These findings support the concept of PAI-1 as an immune-aging checkpoint rather than a disease-specific biomarker alone (Figure 1).

Accordingly, herein, we provide a deeper discussion of PAI-1 biology, a clearer immunoaging rationale, comprehensive preclinical and clinical evidence, and a comparative summary of available PAI-1 inhibitors, with particular focus on TM5614.

## 2. Molecular Biology and Regulation of PAI-1

PAI-1 is a member of the serine protease inhibitor (SERPIN) superfamily and is encoded by SERPINE1. The mature protein consists of approximately 379 amino acids and adopts the characteristic SERPIN fold, including a reactive center loop that interacts with target proteases [4,18]. Upon binding to tPA or uPA, PAI-1 undergoes conformational rearrangement and forms a stable inhibitory complex that prevents plasminogen activation [4,18,19].

A distinctive feature of PAI-1 is its conformational plasticity. Active PAI-1 is intrinsically unstable and spontaneously converts into a latent inactive form. Vitronectin stabilizes active PAI-1 in the extracellular matrix and circulation, prolonging its functional half-life and linking fibrinolytic inhibition to matrix biology, cell adhesion, migration, and tissue remodeling [4,18,19].

SERPINE1 expression is regulated by inflammatory cytokines, transforming growth factor-beta (TGF-beta), angiotensin II, oxidative stress, hypoxia, metabolic stress, and cellular senescence pathways [4,11,20,21,22]. TGF-beta is particularly important in fibrotic diseases because it induces PAI-1 transcription while simultaneously promoting fibroblast activation and extracellular matrix deposition. Hypoxia and oxidative stress further increase PAI-1 expression in damaged tissues, tumors, and metabolically stressed organs.

Mechanistically, PAI-1 can influence senescence through extracellular and intracellular pathways. Extracellularly, PAI-1 suppresses plasmin-mediated matrix degradation and alters cell–matrix interactions. Intracellularly, studies in alveolar epithelial cells and other systems suggest that PAI-1 can reinforce p53/p21 signaling and inhibit proteasome-mediated p53 degradation, thereby stabilizing cell-cycle arrest and senescence programs [11]. These mechanisms help explain why PAI-1 is repeatedly implicated in both tissue fibrosis and organismal aging.

## 3. PAI-1, Cellular Senescence, and Immunoaging

Cellular senescence is a stress response characterized by durable cell-cycle arrest, resistance to apoptosis, chromatin remodeling, metabolic reprogramming, and secretion of SASP factors [7,8,9,10]. Senescence initially acts as a tumor-suppressive and wound-healing mechanism, but chronic senescent-cell accumulation promotes sterile inflammation, tissue dysfunction, fibrosis, and age-related disease [7,8,9,10,23].

PAI-1 is one of the most consistent SASP-associated proteins and is increasingly viewed as a functional mediator of senescence rather than a passive marker. PAI-1 expression rises during replicative and stress-induced senescence, while genetic or pharmacological PAI-1 suppression can delay senescence phenotypes in vascular, epithelial, and stromal cells [3,10,11,12,24]. In accelerated-aging klotho-deficient mice, PAI-1 deficiency or suppression delays senescence-associated pathology, protects organ structure and function, and prolongs survival [25,26].

The relevance of PAI-1 to human aging is supported by a rare loss-of-function SERPINE1 mutation in the Old Order Amish population. Carriers exhibit reduced PAI-1 activity and favorable healthspan-related traits, including improved metabolic parameters and longer lifespan, supporting the possibility that partial reduction of PAI-1 activity may influence human biological aging [27].

The concept of immunoaging is central to understanding the therapeutic rationale for PAI-1 inhibition. Aging is associated with impaired immune surveillance, reduced clearance of senescent cells, chronic activation of myeloid and innate immune pathways, and a persistent inflammatory milieu. PAI-1 may amplify this state by sustaining SASP signaling, promoting monocyte/macrophage recruitment, altering efferocytosis, increasing stromal stiffness, and reinforcing pro-fibrotic extracellular matrix remodeling [3,4,5,6,13,14,15,16,17,20].

**Figure 2:**
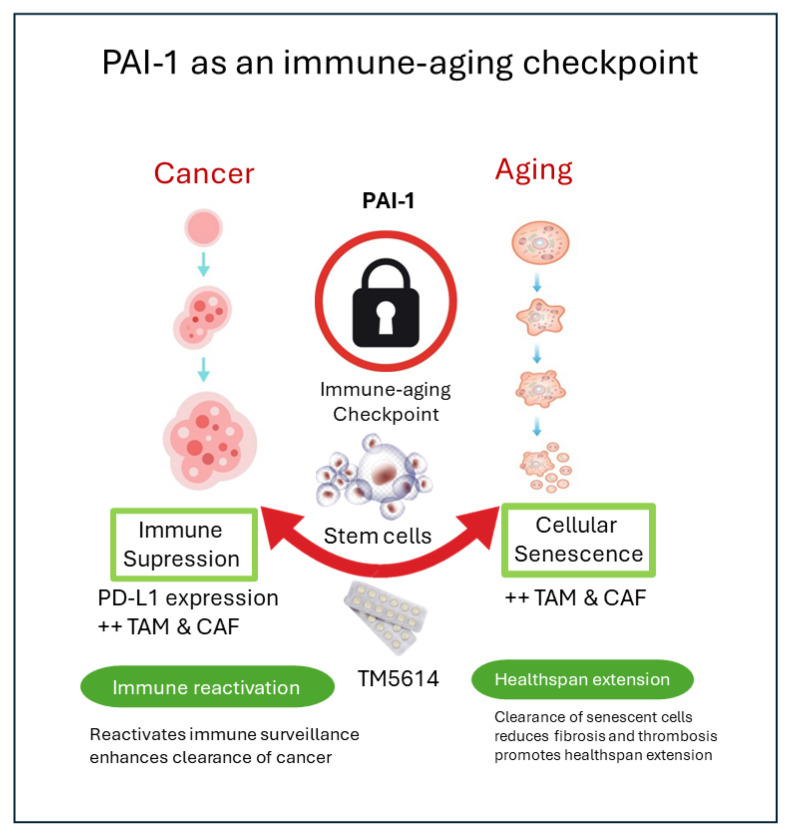
Mechanistic model of PAI-1 as an immune-aging checkpoint. PAI-1 integrates senescence signaling, SASP propagation, macrophage and fibroblast remodeling, PD-L1-associated immune evasion, thrombosis, and fibrosis. PAI-1 inhibition is proposed to reduce pathological matrix retention and immune suppression while improving tissue repair and fibrinolytic balance.

Moreover, senescent cells can upregulate immune checkpoint molecules, including PD-L1, creating a state of local immune escape that resembles tumor immune evasion [13]. PAI-1-rich microenvironments may therefore contribute to failure of immune clearance in both senescent tissues and malignancy. This provides a mechanistic rationale for combining PAI-1 inhibition with immune checkpoint blockade in selected cancers and for investigating PAI-1 inhibition as a broader strategy to improve tissue immune surveillance in aging (Figure 2).

## 4. Discovery and Development of Small-Molecule PAI-1 Inhibitors

Development of PAI-1 inhibitors has been challenging because PAI-1 is a conformationally dynamic SERPIN rather than a conventional enzyme with a stable catalytic pocket. Early compounds demonstrated proof of concept but often faced limitations related to potency, selectivity, pharmacokinetics, or translational safety. Nevertheless, multiple small molecules have been developed and used in preclinical disease models, including tiplaxtinin (PAI-039), TM5275, TM5441, TM5509, and TM5614 [18,24,28,29,30,31,32,33,34,35,36].

Tiplaxtinin (PAI-039) was one of the earliest orally active small-molecule PAI-1 antagonists and showed antithrombotic and profibrinolytic effects in preclinical thrombosis models [28,29]. The TM-series compounds were developed through structure-guided and in silico approaches based on the crystal structure of human PAI-1. Screening of approximately two million virtual compounds identified candidate molecules with predicted PAI-1 inhibitory activity; subsequent synthesis and optimization of more than 1400 derivatives generated compounds including TM5275, TM5441, TM5509, and TM5614 [36].

**Figure 3:**
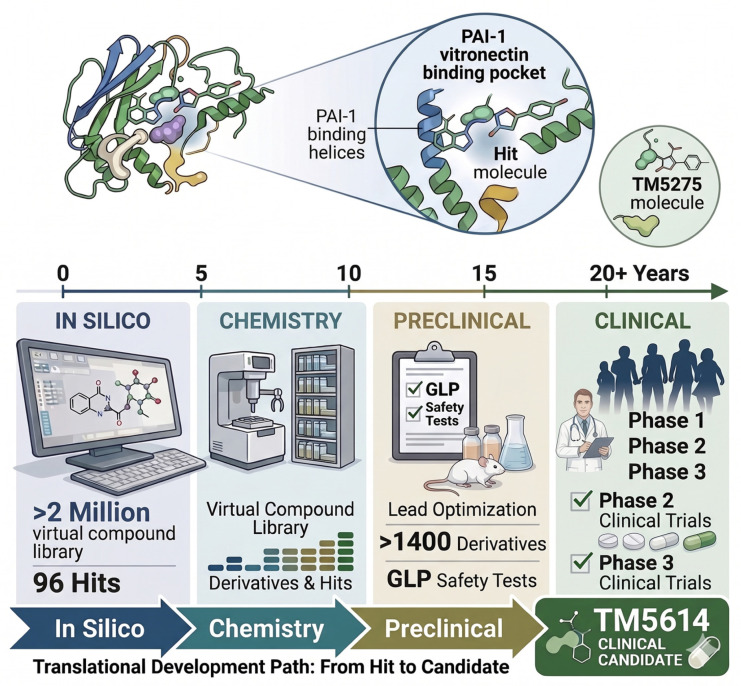
Schematic of the In Silico to Preclinical Workflow. Detailed view of the discovery process starting from a virtual compound library (>2 Million) yielding 96 hits. The process includes chemical synthesis of a virtual compound library and lead optimization of >1400 derivatives, followed by GLP safety testing in animal models.

TM5614 is an orally available investigational small molecule selected from this optimization program (Figure 3) [36,37].

Its development is notable because it advanced from academic drug discovery through preclinical testing, formulation, and clinical evaluation (Table 1).

In contrast to tool compounds used primarily in preclinical systems, TM5614 has been evaluated in human studies, including chronic myeloid leukemia, malignant melanoma, COVID-19-associated pneumonia, and an ongoing or protocolized non-small-cell lung cancer program [38,39,40,41].

## 5. Preclinical Evidence Supporting PAI-1 Inhibition

Representative preclinical studies provide important mechanistic support for PAI-1 inhibition across thrombotic, fibrotic, inflammatory, oncologic, and aging-related disease models. These models are important because they demonstrate that PAI-1 is not only associated with disease severity, but may actively contribute to pathological tissue remodeling, impaired fibrinolysis, senescence persistence, and immune-regulatory dysfunction. Across these experimental systems, pharmacological or genetic reduction of PAI-1 activity has been associated with reduced thrombosis, enhanced fibrinolytic activity, attenuation of fibrosis, suppression of inflammatory remodeling, improved vascular function, reduction of senescence-associated markers, and modulation of cancer-related stromal and immune pathways.

The preclinical evidence also supports the concept that PAI-1 inhibition may have effects extending beyond classical coagulation biology. By restoring aspects of extracellular matrix turnover, reducing pathological stromal activation, and altering senescence-associated signaling, PAI-1 inhibition may influence several disease processes that commonly overlap in aging tissues. These findings provide a mechanistic foundation for the clinical development of TM5614 and related compounds while also emphasizing the need to distinguish between evidence generated using earlier preclinical inhibitors and evidence obtained with clinically evaluated compounds.

### 5.1. Thrombosis and Vascular Aging

Because PAI-1 suppresses fibrinolysis by inhibiting tissue-type and urokinase-type plasminogen activators, excessive PAI-1 activity contributes to a prothrombotic biological state. This is particularly relevant in aging, obesity, diabetes, cardiovascular disease, metabolic dysfunction, and inflammatory conditions, where increased PAI-1 expression may impair fibrin clearance and promote vascular complications [2,21,22,42,43]. In these settings, elevated PAI-1 may contribute not only to thrombosis, but also to endothelial dysfunction, impaired nitric oxide signaling, vascular stiffness, and chronic inflammatory remodeling.

Preclinical studies using tiplaxtinin and TM-series compounds have demonstrated that pharmacological PAI-1 inhibition can exert antithrombotic and vascular-protective effects in experimental settings [28,29,31,32]. Tiplaxtinin provided early proof-of-concept that orally active PAI-1 antagonism could enhance fibrinolytic activity and reduce thrombus formation in experimental thrombosis models [28,29]. These findings established PAI-1 as a druggable target in thrombotic disease biology.

Beyond clot formation itself, TM5441 attenuated hypertension-associated vascular remodeling and senescence in an L-NAME model [31]. This finding is important because it suggests that PAI-1 inhibition may influence vascular aging through mechanisms that extend beyond direct effects on fibrinolysis. By reducing vascular senescence, remodeling, and dysfunction, PAI-1 inhibition may help modify the chronic vascular changes that accompany aging and cardiometabolic disease. These findings support the broader interpretation that PAI-1 contributes to a vascular aging phenotype involving thrombosis, impaired endothelial function, and tissue remodeling (Table 2).

### 5.2. Fibrosis and Extracellular Matrix Remodeling

PAI-1 promotes fibrosis primarily by limiting plasmin-mediated extracellular matrix degradation and by interacting with TGF-beta-driven fibroblast activation. Under physiological conditions, the plasminogen activation system contributes to tissue repair by regulating fibrin clearance and matrix turnover. However, when PAI-1 is persistently elevated, this balance shifts toward matrix accumulation, fibroblast persistence, tissue stiffness, and progressive organ dysfunction. This mechanism is particularly relevant in chronic kidney, liver, pulmonary, intestinal, and metabolic fibrotic diseases.

TM5275 and TM5441 reduced renal fibrosis and inflammatory markers in diabetic nephropathy models without obvious bleeding complications in the reported preclinical setting [30]. These findings suggest that PAI-1 inhibition may reduce both fibrotic remodeling and inflammatory injury in metabolically stressed organs. The diabetic nephropathy model is especially relevant because it links PAI-1 to a disease state characterized by hyperglycemia, endothelial dysfunction, inflammation, and progressive extracellular matrix deposition.

In liver fibrosis models associated with metabolic syndrome, TM5275 attenuated fibrotic progression by suppressing hepatic stellate cell activation and collagen synthesis [34]. This supports the concept that PAI-1 inhibition can affect fibrogenic cell behavior, not only extracellular proteolysis. In chronic intestinal fibrosis, oral TM5275 ameliorated fibrotic pathology in a colitis-associated model, further suggesting that the anti-fibrotic effects of PAI-1 inhibition may apply across multiple tissue compartments [35]. Taken together, these findings indicate that PAI-1 inhibition may restore a more favorable balance between matrix deposition and matrix degradation while also reducing inflammation-associated fibrotic remodeling.

### 5.3. Senescence, Organ Dysfunction, and Longevity Biology

The senescence-related effects of PAI-1 inhibition are supported by both genetic and pharmacological models. PAI-1 is repeatedly identified as a senescence-associated factor, and its expression increases in response to cellular stress, DNA damage, oxidative injury, inflammatory signaling, and TGF-beta activation. In this context, PAI-1 may contribute to maintenance of the senescent phenotype by reinforcing extracellular matrix remodeling, inflammatory signaling, and cell-cycle arrest pathways.

In klotho-deficient mice, PAI-1-regulated extracellular proteolysis influenced senescence burden, organ structure, function, and survival [26]. This model is particularly important because it links PAI-1 activity to systemic aging-like pathology rather than a single organ-specific disease process. The improvement of organ function and survival in this setting supports the concept that PAI-1 is not simply a biomarker of aging-associated damage, but may participate functionally in the progression of aging phenotypes.

Pharmacological studies further support this concept. TM5441 protected against stress-induced and aging-associated senescence in cardiovascular cell systems and in vivo models [24,32]. These findings suggest that PAI-1 inhibition can influence cellular senescence programs and vascular aging biology. In addition, a novel PAI-1 inhibitor prevented aging-related skeletal muscle fiber atrophy, suggesting that PAI-1 inhibition may also be relevant to sarcopenia-related tissue decline [44]. Although these findings remain preclinical, they support the broader geroscience rationale that PAI-1 inhibition may affect multiple aging-related tissues through shared mechanisms involving senescence, inflammation, proteolysis, and tissue repair.

### 5.4. Cancer Microenvironment and Immune Evasion

In cancer, PAI-1 biology is complex and context-dependent, but elevated PAI-1 expression is frequently associated with aggressive tumor behavior, stromal remodeling, angiogenesis, macrophage recruitment, epithelial–mesenchymal transition, treatment resistance, and poor prognosis [14,17]. These associations suggest that PAI-1 may contribute to the tumor microenvironment as a regulator of cell migration, extracellular matrix remodeling, stromal activation, and immune suppression.

PAI-1 may also reinforce immunosuppressive tumor microenvironments by promoting interactions among tumor cells, tumor-associated macrophages, cancer-associated fibroblasts, and extracellular matrix components. In this context, PAI-1 inhibition is not necessarily expected to act as a direct cytotoxic therapy. Rather, it may function as a microenvironment-modifying strategy that improves immune surveillance, reduces stromal barriers, and enhances sensitivity to other anticancer treatments.

This concept provides a translational rationale for combining PAI-1 inhibition with immune checkpoint blockade, particularly in tumors resistant to PD-1/PD-L1-targeted therapy. The melanoma and NSCLC clinical programs with TM5614 are based on this tumor-microenvironment rationale [39,40]. These studies reflect the hypothesis that reducing PAI-1 activity may help reverse immune-resistant stromal conditions and improve responsiveness to immune checkpoint inhibition.

**Figure 4:**
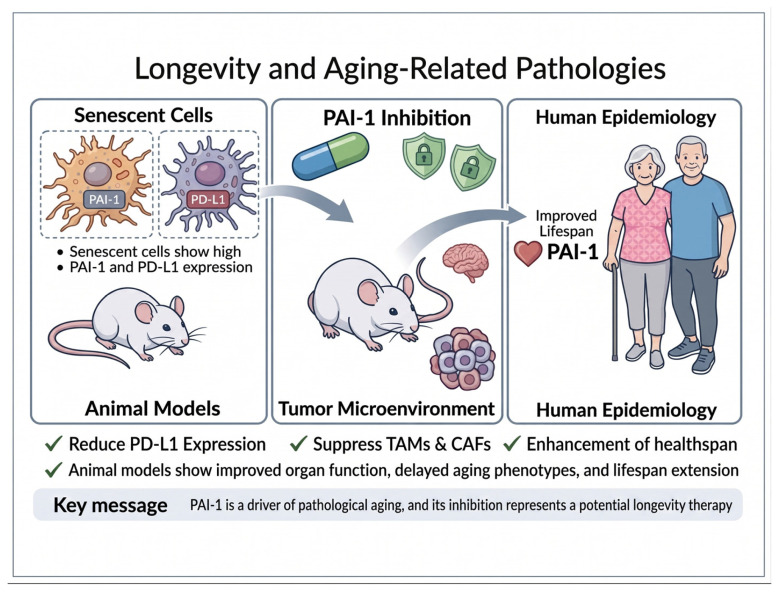
Mechanistic Role of PAI-1 in Cellular Senescence and Organismal Aging. (**Left**) Senescent cells exhibit high expression of PAI-1 and PD-L1. (**Center**) PAI-1 inhibition modulates the tumor microenvironment. (**Right**) Evidence from animal models and human epidemiology suggests that PAI-1 inhibition enhances healthspan and improves organ function.

PAI-1 therefore integrates several biological processes that are highly relevant to age-associated pathology and cancer progression. Through its effects on fibrinolysis, extracellular matrix turnover, stromal remodeling, senescence-associated secretory signaling, macrophage and fibroblast activity, and immune regulation, PAI-1 may contribute to a tissue environment characterized by chronic inflammation, impaired repair, fibrosis, thrombosis, reduced immune surveillance, and therapeutic resistance. This mechanistic framework is summarized in Figure 4.

## 6. TM5614: Pharmacological Characteristics and Clinical Translation

TM5614 is a small-molecule PAI-1 inhibitor derived from structure-guided medicinal chemistry. It is orally administered and was selected from a large optimization program because of its inhibitory activity, pharmacological profile, and translational suitability [36,40]. A clinically important feature is that TM5614 aims to reduce pathological PAI-1 activity without complete abolition of hemostatic control. Nevertheless, because PAI-1 is integral to fibrinolytic balance, bleeding surveillance remains necessary in all clinical development programs.

### 6.1. Chronic Myeloid Leukemia

The CML rationale is based on the role of PAI-1 in hematopoietic stem-cell retention within protective bone marrow niches. PAI-1 can promote niche retention through mechanisms involving intracellular protease regulation and TGF-beta-related signaling [15,16]. In chronic-phase CML, combining TM5614 with tyrosine kinase inhibitors was investigated to mobilize quiescent leukemic stem cells and increase susceptibility to therapy. A phase II study reported that TM5614 plus TKI therapy was well tolerated and induced deep molecular response in more patients than expected with stand-alone TKI treatment, supporting further investigation of this strategy [38].

### 6.2. Immune-Checkpoint-Refractory Malignant Melanoma

The malignant melanoma program directly addresses the proposed relationship between PAI-1, tumor-microenvironment remodeling, immune escape, and resistance to immune checkpoint therapy. Anti-PD-1 therapy has transformed the treatment of advanced melanoma; however, a substantial proportion of patients develop primary or acquired resistance. In this context, resistance is not determined only by tumor-intrinsic mechanisms, but also by the surrounding immune and stromal microenvironment, including myeloid-cell infiltration, fibroblast activation, extracellular matrix remodeling, angiogenic signaling, and impaired cytotoxic immune-cell access.

PAI-1 is biologically relevant to this setting because elevated PAI-1 activity may support a tumor microenvironment characterized by stromal activation, macrophage recruitment, epithelial–mesenchymal transition, and immune suppression. Therefore, inhibition of PAI-1 may help convert an immunologically resistant microenvironment into one that is more permissive to immune checkpoint blockade. This rationale is particularly relevant in anti-PD-1-refractory disease, where simply continuing immune checkpoint inhibition may be insufficient unless additional microenvironmental resistance mechanisms are modified.

In the multicenter phase II TM5614-MM study, TM5614 was combined with nivolumab in patients with anti-PD-1-refractory malignant melanoma [39]. The study evaluated whether pharmacological PAI-1 inhibition could enhance responsiveness to nivolumab by targeting immunosuppressive stromal and myeloid pathways. The published trial supports the feasibility and clinical relevance of this combination approach while also indicating that future studies should incorporate biomarker-guided patient selection. Such biomarkers may include PAI-1 pathway activity, immune-cell infiltration patterns, stromal signatures, and markers of immune checkpoint resistance.

Overall, the melanoma data provide important clinical support for the concept that PAI-1 inhibition may be most useful as a microenvironment-modifying therapy rather than as a direct anticancer monotherapy. This interpretation aligns with the broader hypothesis that PAI-1 acts as a mediator of immune evasion and stromal resistance in select tumor contexts.

### 6.3. Non-Small-Cell Lung Cancer

The non-small-cell lung cancer program extends the same tumor-microenvironment rationale to another major solid tumor type in which immune checkpoint inhibitors are widely used but therapeutic resistance remains common. In previously treated NSCLC, nivolumab and related immune checkpoint inhibitors can provide meaningful benefit, but many tumors remain unresponsive or eventually progress. Resistance may involve insufficient immune-cell infiltration, expansion of immunosuppressive myeloid populations, stromal exclusion, extracellular matrix remodeling, hypoxia, and inflammatory pathways that limit effective antitumor immunity.

PAI-1 may contribute to several of these resistance mechanisms. By influencing extracellular matrix turnover, tumor-cell migration, macrophage recruitment, fibroblast activation, and immune-regulatory signaling, PAI-1 can help shape a tumor microenvironment that is less responsive to immune-mediated tumor clearance. Therefore, combining a PAI-1 inhibitor with immune checkpoint blockade is a rational strategy to test whether modification of the stromal and myeloid microenvironment can improve immunotherapy responsiveness.

An investigator-initiated phase II protocol has been reported for nivolumab plus TM5614 in previously treated NSCLC [40]. This program builds on the concept that PAI-1 inhibition may enhance the activity of immune checkpoint blockade by reducing PAI-1-associated immune resistance mechanisms. Although the reported study is primarily a protocol/design report rather than definitive efficacy evidence, it is important because it shows that the biological rationale for TM5614 has progressed into prospective clinical evaluation in lung cancer.

The NSCLC program also helps broaden the translational relevance of TM5614 beyond melanoma. If PAI-1 inhibition can reproducibly modify immunosuppressive tumor microenvironments across different cancer types, it may become a useful adjunctive strategy for select patients receiving immune checkpoint inhibitors. Future clinical interpretation will require careful integration of clinical outcomes with immune and stromal biomarkers.

### 6.4. COVID-19-Associated Pneumonia and Inflammatory Thrombosis

PAI-1 has also been implicated in inflammatory thrombosis, endothelial dysfunction, impaired fibrinolysis, and fibrotic remodeling during severe inflammatory states. These processes became particularly relevant during COVID-19, where pulmonary inflammation, endothelial injury, microvascular thrombosis, and post-inflammatory fibrotic changes were recognized as major contributors to disease severity. Because PAI-1 suppresses fibrinolysis and may promote thrombo-inflammatory tissue injury, pharmacological PAI-1 inhibition was considered a plausible therapeutic approach in this setting.

In mild-to-moderate COVID-19-associated pneumonia, phase IIa/IIb evaluation of TM5614 investigated whether inhibition of PAI-1 could reduce thrombotic, inflammatory, or fibrotic progression [41]. The clinical rationale was not limited to prevention of clot formation. Rather, TM5614 was evaluated within a broader thrombo-inflammatory framework involving endothelial dysfunction, impaired fibrin clearance, inflammatory remodeling, and possible progression toward lung fibrosis.

The study reported feasibility and safety observations, providing useful human translational information for TM5614 in an acute inflammatory disease context [41]. However, the findings do not establish TM5614 as standard therapy for COVID-19. Instead, the study should be interpreted as supportive clinical experience showing that PAI-1 inhibition can be evaluated in humans under inflammatory and thrombo-fibrotic conditions. This information is relevant for future development in diseases where inflammation, thrombosis, endothelial injury, and fibrosis overlap (Table 3).

More broadly, the COVID-19 study contributes to the safety and translational evidence base for TM5614. It supports continued investigation of PAI-1 inhibition in carefully selected thrombo-inflammatory or fibrotic diseases while reinforcing the need for appropriate monitoring of bleeding risk, coagulation/fibrinolysis parameters, disease stage, and patient-specific risk factors.

## 7. PAI-1 in Cancer and Fibrotic Disease

High PAI-1 expression has been observed in many malignancies and is often associated with invasive behavior, metastatic potential, treatment resistance, stromal activation, angiogenesis, and poor clinical outcomes [14,17]. Although PAI-1 biology is context-dependent and may vary according to tumor type, disease stage, and microenvironmental composition, the weight of translational evidence supports its role as an active mediator of tumor–stroma interaction rather than a simple fibrinolytic factor.

PAI-1 influences the tumor microenvironment through several overlapping mechanisms. These include regulation of cell adhesion and migration, extracellular matrix remodeling, macrophage recruitment, fibroblast activation, angiogenic signaling, and possible interaction with immune checkpoint biology [13,14,17,39,40]. Through these mechanisms, elevated PAI-1 may contribute to a tumor niche that supports invasion, stromal protection, immune escape, and resistance to therapy. This may be particularly relevant in tumors with abundant fibrotic stroma or myeloid-cell infiltration, where physical and immunological barriers limit effective antitumor immunity.

These mechanisms explain why PAI-1 inhibition may be most useful not as a direct cytotoxic strategy, but as a microenvironment-modifying approach. By reducing stromal activation, improving extracellular matrix turnover, and potentially attenuating immunosuppressive myeloid and fibroblast pathways, PAI-1 inhibition may enhance the effects of targeted therapy, chemotherapy, or immune checkpoint blockade. This concept is supported by the clinical development of TM5614 in combination with nivolumab in melanoma and NSCLC, where the therapeutic rationale is based on modifying PAI-1-associated immune resistance rather than directly killing tumor cells [39,40].

In fibrotic disease, PAI-1 provides a mechanistic link between TGF-beta signaling, impaired matrix degradation, and progressive tissue stiffness. Under normal conditions, the plasminogen activation system contributes to extracellular matrix turnover and tissue repair. However, persistent PAI-1 elevation suppresses plasmin-mediated matrix degradation and promotes accumulation of fibrin and extracellular matrix components. This creates a tissue environment characterized by stiffness, inflammatory persistence, fibroblast activation, and progressive organ dysfunction.

Preclinical models in the kidney, liver, lung, and intestine support anti-fibrotic effects of PAI-1 inhibition [30,34,35]. These findings suggest that PAI-1 inhibition may help restore the balance between matrix deposition and matrix degradation. They also indicate that PAI-1 activity may serve as a useful marker of pathway engagement in fibrotic diseases. Future clinical evaluation should therefore focus on carefully selected patient populations in which PAI-1 expression, fibrinolytic imbalance, or fibrotic remodeling is biologically relevant and measurable.

## 8. PAI-1 Inhibition and Longevity

Geroscience seeks to identify interventions that target shared biological mechanisms underlying multiple age-related diseases rather than treating each disease as an isolated condition [23]. PAI-1 is an attractive candidate target within this framework because it lies at the intersection of cellular senescence, inflammaging, fibrosis, thrombosis, metabolic dysfunction, vascular aging, and impaired tissue regeneration [3,5,6,23,25,26,27].

The longevity rationale for PAI-1 inhibition is supported by converging lines of evidence. Genetic reduction of PAI-1 activity improves phenotypes in accelerated-aging models, pharmacological inhibition reduces senescence-associated and fibrotic changes in several organs, and human SERPINE1 loss-of-function carriers show favorable healthspan-associated traits [24,26,27,32,44]. These findings suggest that PAI-1 may participate functionally in aging biology rather than merely reflecting downstream tissue damage.

Mechanistically, PAI-1 may contribute to aging by reinforcing senescence-associated secretory signaling, suppressing extracellular matrix turnover, promoting inflammatory and fibrotic remodeling, impairing vascular homeostasis, and reducing tissue repair capacity. These effects are not limited to one organ system. Instead, they may operate across vascular, metabolic, musculoskeletal, pulmonary, and immune-related tissues, which explains why PAI-1 has emerged as a potential geroscience target.

However, longevity translation requires caution. PAI-1 has physiological roles in hemostasis, tissue repair, and wound healing; therefore, complete or poorly timed inhibition may be undesirable in some contexts. The goal of therapeutic development should not be indiscriminate suppression of PAI-1, but controlled pathway modulation in patients or disease states where pathological PAI-1 activity contributes to tissue dysfunction. Future studies should define optimal dosing, patient selection, biomarkers, duration of therapy, and safety monitoring, including bleeding risk, wound healing, and interactions with anticoagulants or antiplatelet agents.

## 9. Safety Considerations and Translational Limitations

Because PAI-1 is a key inhibitor of fibrinolysis, bleeding risk is the most obvious theoretical safety concern. Available preclinical and early clinical studies have not uniformly shown prohibitive bleeding signals, but this should not be interpreted as absence of risk. Clinical trials should systematically monitor bleeding events, coagulation and fibrinolysis parameters, concomitant anticoagulant or antiplatelet exposure, liver and renal function, wound healing, and patient-specific thrombotic versus hemorrhagic risk.

A second limitation is biological context. PAI-1 may exert different effects depending on disease stage, tissue type, cellular source, inflammatory state, and the balance between protective wound repair and pathological remodeling. In early tissue injury, some PAI-1 activity may contribute to hemostatic control and repair. In chronic disease, persistent PAI-1 elevation may instead promote fibrosis, thrombosis, immune suppression, and impaired regeneration. This context dependence is particularly important when considering long-term or preventive applications.

In cancer, PAI-1 biology is also complex. PAI-1 may influence tumor invasion, stromal remodeling, angiogenesis, macrophage recruitment, immune escape, and response to therapy in ways that differ by tumor type and microenvironmental state. Therefore, future clinical trials should not rely only on disease diagnosis but should incorporate biomarker strategies to identify patients most likely to benefit. Relevant biomarkers may include circulating PAI-1, SERPINE1 expression, SASP signatures, fibrosis markers, immune-cell profiling, PD-L1 or immune checkpoint context, and pharmacodynamic measures of fibrinolytic balance.

A third limitation is that not all PAI-1 inhibitors are equivalent. Tool compounds such as tiplaxtinin, TM5275, and TM5441 provide important mechanistic and preclinical evidence, but their results cannot be directly generalized to all clinical settings. Clinical conclusions should be drawn primarily from compounds tested in humans, particularly TM5614. Accordingly, preclinical evidence from earlier tool compounds should be interpreted separately from clinical evidence generated with TM5614.

## 10. Future Perspectives

Targeting PAI-1 represents a promising strategy for diseases driven by shared thrombotic, fibrotic, inflammatory, stromal, and senescence-related mechanisms. The therapeutic opportunity is broad, but successful development will require precision rather than generalized PAI-1 suppression. PAI-1 inhibition is most likely to be useful in settings where pathway activation is biologically relevant, measurable, and linked to disease progression or treatment resistance.

Future studies should prioritize biomarker-defined patient selection, pharmacodynamic confirmation of PAI-1 pathway inhibition, rational combination strategies, and longitudinal assessment of senescence and immune-aging markers. Combination approaches may be particularly important, including pairing PAI-1 inhibition with tyrosine kinase inhibitors, immune checkpoint inhibitors, anti-fibrotic therapies, or metabolic interventions. Such strategies may allow PAI-1 inhibition to function as a disease-modifying or microenvironment-modifying therapy rather than as a standalone intervention.

Safety monitoring will remain essential. Future clinical programs should carefully assess bleeding, wound repair, fibrinolytic balance, and interactions with anticoagulants or antiplatelet agents. Long-term studies should also evaluate whether partial inhibition of pathological PAI-1 activity can preserve physiological hemostatic functions while reducing chronic thrombo-inflammatory and fibrotic remodeling.

As TM5614 progresses through clinical development, it provides a rare example of an academically discovered small molecule moving from structural biology and medicinal chemistry into human translational testing. Its ultimate value will depend on whether PAI-1 inhibition can produce clinically meaningful benefits in diseases where senescence, fibrosis, immune evasion, and thrombosis converge.

**Figure 5:**
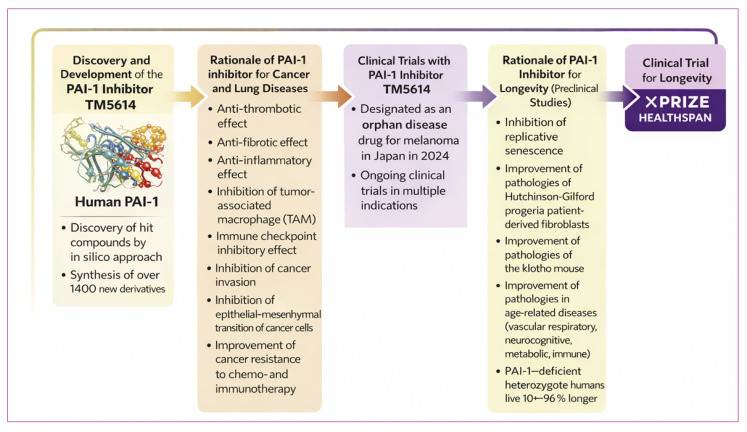
Translational development and therapeutic rationale of the PAI-1 inhibitor TM5614. This schematic illustrates the discovery and clinical translation of the small-molecule PAI-1 inhibitor TM5614. Structure-guided drug discovery based on the crystal structure of human PAI-1 enabled in silico screening and medicinal chemistry optimization, generating over 1400 derivatives and identifying TM5614 as a lead candidate. Preclinical studies demonstrate that PAI-1 inhibition exerts anti-thrombotic, anti-fibrotic, and anti-inflammatory effects while modulating the tumor microenvironment by reducing tumor-associated macrophages, suppressing epithelial–mesenchymal transition, and decreasing immune checkpoint signaling. TM5614 has advanced to clinical trials for cancer and lung diseases and received orphan drug designation for melanoma in Japan in 2024. Experimental and epidemiological evidence further suggests that reduced PAI-1 activity may influence aging biology and extend healthspan, supporting investigation of PAI-1 inhibition as a potential longevity-targeting therapy.

Preclinical data across thrombosis, fibrosis, vascular aging, metabolic disease, muscle aging, and cancer models support the therapeutic rationale for PAI-1 inhibition. Clinical studies of TM5614 in chronic myeloid leukemia, melanoma, NSCLC, and COVID-19-associated pneumonia provide early translational evidence and justify further investigation. This translational pathway is summarized in Figure 5.

## 11. Conclusions

PAI-1 has emerged as a central molecular regulator linking fibrinolysis, cellular senescence, immunoaging, fibrosis, thrombosis, and cancer microenvironment biology. The framework presented here positions PAI-1 as an immune-aging checkpoint: a targetable node through which senescent and diseased tissues maintain inflammation, immune evasion, matrix remodeling, and impaired repair.

Future progress will depend on biomarker-guided clinical trials, rational combination therapy, and careful safety monitoring, particularly for bleeding and wound-healing risks.

## Figures and Tables

**Table 1:** Representative PAI-1 inhibitors and development status.

Compound	Type/Generation	Representative Evidence	Developmental Relevance
Tiplaxtinin (PAI-039)	Early orally active small-molecule PAI-1 antagonist	Reduced thrombosis and enhanced fibrinolysis in preclinical arterial/venous thrombosis models [28,29].	Important proof-of-concept compound; primarily preclinical.
TM5275	Orally active TM-series PAI-1 inhibitor	Reported anti-fibrotic effects in diabetic nephropathy, liver fibrosis, intestinal fibrosis, and asthma-related remodeling models [30,34,35].	Widely used preclinical compound supporting anti-fibrotic rationale.
TM5441	TM-series PAI-1 inhibitor	Attenuated vascular senescence, hypertension-associated remodeling, high-fat-diet-induced metabolic injury, and stress-induced cellular senescence [24,31,32,33].	Preclinical compound supporting anti-senescent and cardiometabolic rationale.
TM5509	Optimized TM-series candidate	Identified during medicinal chemistry optimization of PAI-1 inhibitors [36].	Intermediate/lead-optimization compound; relevant to lineage of TM5614.
TM5614/RS5614	Orally available investigational PAI-1 inhibitor	Evaluated clinically with TKI therapy in chronic-phase CML, with nivolumab in anti-PD-1-refractory melanoma, in COVID-19 pneumonia, and in NSCLC trial design [38,39,40,41].	Most clinically advanced compound discussed in this review.

**Table 2:** Representative preclinical evidence supporting PAI-1 inhibition.

Disease Area	Model/Compound	Main Reported OUTCOME	Translational Implication
	Tiplaxtinin/PAI-039 in thrombosis models	Antithrombotic and profibrinolytic effects [28,29]	Supports fibrinolytic rationale; requires bleeding-risk monitoring in translation.
Vascular aging	TM5441 in L-NAME-induced hypertension	Reduced vascular senescence and cardiovascular remodeling [31]	Links PAI-1 inhibition to vascular aging biology.
Diabetic nephropathy	TM5275 and TM5441 in STZ-induced diabetic mice	Reduced kidney fibrosis and inflammatory markers [30]	Supports anti-fibrotic and anti-inflammatory potential.
Liver fibrosis	TM5275 in metabolic syndrome-related fibrosis models	Reduced hepatic stellate cell activation and collagen synthesis [34]	Supports application in metabolic/fibrotic liver disease.
Intestinal fibrosis	TM5275 in chronic colitis-associated fibrosis	Ameliorated fibrotic pathology [35]	Suggests broader tissue fibrosis relevance.
Accelerated aging	PAI-1 suppression/deficiency in klotho mice	Improved organ structure/function and prolonged survival [26]	Provides longevity-biology rationale.
Muscle aging	PAI-1 inhibitor in aging-related muscle atrophy model	Prevention of muscle fiber atrophy [44]	Supports geroscience and sarcopenia relevance.
Cancer microenviroment	PAI-1 inhibition rationale in melanoma/NSCLC	Modulation of stromal and immune resistance pathways [14,17,39,40]	Supports combination with immune checkpoint blockade.

**Table 3:** Representative clinical development of TM5614.

Indication	Therapeutic Concept	Clinical Evidence	Key Interpretation
Chronic-phase CML	Mobilize leukemic stem cells from bone marrow niche and enhance TKI response.	TM5614 plus TKI phase II study reported tolerability and increased deep molecular response [38].	Supports stem-cell niche strategy; requires longer follow-up for treatment-free remission impact.
Anti-PD-1-refractory melanoma	Modify PAI-1-rich immunosuppressive microenvironment and restore checkpoint sensitivity.	Multicenter phase II TM5614 plus nivolumab trial published in 2024 [39].	Provides proof-of-concept for immuno-oncology combination.
Previously treated NSCLC	Enhance nivolumab activity by targeting PAI-1-mediated immune resistance.	Investigator-initiated phase II protocol/design reported [40].	Translationally aligned with melanoma rationale.
COVID-19 pneumonia	Reduce inflammatory thrombosis/fibrosis and endothelial injury biology.	Randomized double-blind placebo-controlled trial reported in 2024 [41].	Informative for safety and thrombo-inflammatory biology; not definitive standard-of-care evidence.

## Data Availability

No new data were created or analyzed in this study.
